# Prevalence of Cytopenia in the General Population—A National Health and Nutrition Examination Survey Analysis

**DOI:** 10.3389/fonc.2020.579075

**Published:** 2020-11-20

**Authors:** Naomi Alpert, Joseph L. Rapp, John Mascarenhas, Eileen Scigliano, Douglas Tremblay, Bridget K. Marcellino, Emanuela Taioli

**Affiliations:** ^1^ Institute for Translational Epidemiology, Icahn School of Medicine at Mount Sinai, New York, NY, United States; ^2^ Department of Medicine, Division of Hematology and Medical Oncology, Icahn School of Medicine at Mount Sinai, New York, NY, United States; ^3^ Tisch Cancer Institute, Icahn School of Medicine at Mount Sinai, New York, NY, United States; ^4^ Department of Environmental Medicine and Public Health, Icahn School of Medicine at Mount Sinai, New York, NY, United States

**Keywords:** anemia, neutropenia, thrombocytopenia, nationally representative survey, clinical determinants

## Abstract

**Background:**

Cytopenia, a reduced count of blood cells manifesting as anemia, neutropenia, and/or thrombocytopenia is frequently associated with other medical conditions. However, a cytopenia may not be accompanied by a known determinant and in some of these cases, may be a precursor to pre-malignancies or hematologic cancers. Little is known about the prevalence of these unexplained cytopenias and their distribution in the population.

**Materials and Methods:**

The National Health and Nutrition Examination Survey (NHANES) from 1999 to 2002 was used to identify those with a cytopenia in the general population. Those without an identifiable determinant in the NHANES were classified as having unexplained cytopenia. Weighted frequencies were examined to assess the prevalence of unexplained cytopenia in the population. Distribution of blood counts comparing those with unexplained cytopenia to the general population was examined. Multivariable logistic regression was conducted to assess the association between unexplained cytopenia and demographic factors.

**Results:**

Of the 7,962 people in the sample, 236 (2.0%) had any cytopenia and 86 (0.9%) had an unexplained cytopenia. Approximately 43% of all cytopenias were not accompanied by a clinical determinant. Unexplained cytopenia was more common in men (1.1%) than in women (0.7%) and in Non-Hispanic Black participants (3.4%). Among those with an unexplained cytopenia, the majority (74.8%) manifested as neutropenia. Compared to those with no cytopenia, those with unexplained cytopenia were significantly less likely to be female, have body mass index ≥30 kg/m^2^, and work in the service industry, and were significantly more likely to be non-Hispanic Black.

**Conclusions:**

This is the first study to examine the prevalence of unexplained cytopenia in a nationally representative sample and may serve as a baseline for comparison with other populations. Future research to identify risk factors for development of malignant hematological disorders among those with unexplained cytopenia is warranted.

## Introduction

Cytopenia, defined by a reduced number of blood cells manifesting as either anemia, neutropenia, and/or thrombocytopenia (1), may be associated with multiple conditions, including cancer, bone marrow suppression from chemotherapy or radiotherapy, as well as pregnancy, nutrient deficiencies, liver disease, hypersplenism, and renal insufficiency (2–5). However, for some patients, the presence of a cytopenia is not accompanied by a known determinant. There is limited research on the patterns of unexplained cytopenia (UC) in the general population, and little is known about the prevalence and evolution of this hematologic finding over time. Patients with a cytopenia may be asymptomatic with no clinical sequelae or can experience a wide variety of adverse health outcomes including symptoms such as fatigue, weakness, increased infections, or life threatening bleeding (2, 6, 7).

There are cases of cytopenias that do not have clinical repercussions. Benign ethnic neutropenia (8) is one such example, where patients have low neutrophil counts but normal quantities of lymphocytes and other leukocyte subtypes, normal bone marrow morphologic features and cellularity, and no increased risk of infections (9).

Some cases of UC, however, may be precursors to a variety of pre-malignancies as well as certain hematologic cancers. A UC that persists for ≥6 months, without other diagnostic criteria for myelodysplastic syndrome (MDS) and which is not explained by other hematologic disorders, is termed an idiopathic cytopenia of undetermined significance (ICUS) (1). A subset of patients with unexplained cytopenias harbor somatic mutations in myeloid malignancy associated genes in hematopoietic cells, and these cases are termed clonal cytopenias of undetermined significance (CCUS). These patients have a heightened risk of developing hematologic malignancies including MDS, acute myeloid leukemia (AML), myeloproliferative neoplasms (MPN), or other hematologic malignancies and disorders of the hematopoietic system, such as systemic mastocytosis, multiple myeloma, non-Hodgkin lymphoma, aplastic anemia, or paroxysmal nocturnal hemoglobinuria (10). Given that leukemia is the tenth most common cancer in the United States (US) (11), and its prognosis may be dismal, follow-up and surveillance of these patients are of particular importance.

Little is known about the prevalence of UC in the US, and existing research about the prevalence of unexplained anemia has largely focused on older adults (5). The goal of this study was to use the National Health and Nutrition Examination Survey (NHANES), a US population based sample, to estimate the prevalence of cytopenia and UC in the US, as well as to characterize the demographic characteristics associated with UC.

## Materials and Methods

### Data Source

NHANES is a publicly available, cross-sectional, population-based survey administered by the Centers for Disease Control and Prevention (CDC) that aims to evaluate health and nutritional status of the civilian, non-institutionalized US population, using a combination of interviews, physical examinations, and laboratory testing. NHANES data is released in 2-year cycles and utilizes a multi-stage probability sampling design and weighting in order to produce a nationally representative sample at each cycle (12). The NHANES protocol is reviewed annually by the National Center for Health Statistics Ethics Review Board (13).

### Study Population

**Figure 1:**
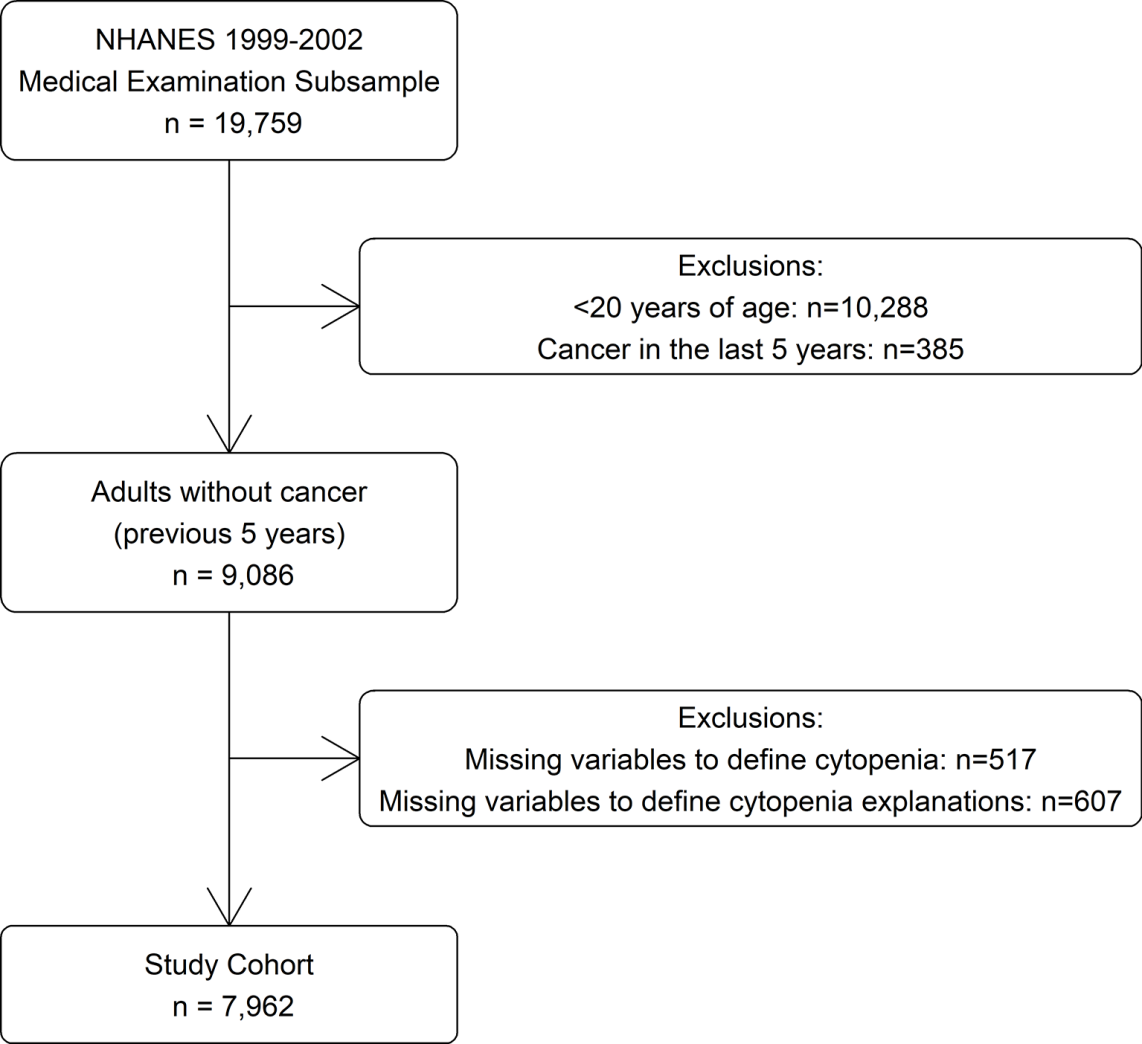
Selection criteria.

The data for this study was derived from the 1999 to 2000 and 2001 to 2002 survey cycles (14), as these were the most recent years in which information was available on all variables used to identify cytopenia and its determinants. Participants were selected from the subsample who were selected for a physical examination (n = 19,759). Those <20 years of age (n = 10,288) were excluded as they were not questioned about cancer history. Those diagnosed with cancer within the last 5 years (n = 385) and those missing laboratory values needed to define cytopenia or possible explanations for cytopenia (n = 1,124) were also excluded, for a study cohort of 7,962 (**Figure 1**).

### Definition and Possible Determinants of Cytopenia

Cytopenia was defined if a participant had at least one of the following: anemia (hemoglobin <11 g/dl) (15), neutropenia (<1,500 neutrophils/µl), or thrombocytopenia (<100,000 platelets/µl), based on recommended criteria for ICUS (1). Although sex-specific cutoffs are sometimes used to define normal hemoglobin levels, these criteria recommend a common cutoff for males and females. Although a full clinical examination to classify definitively all determinants of cytopenia was not available, possible determinants that were measured in NHANES were included for analysis. Iron deficiency was defined if a participant had ≥2 of the following situations: transferrin saturation <15%, serum ferritin concentration <12 ng/ml, and erythrocyte protoporphyrin concentration >1.24 μM. Vitamin B_12_ deficiency was defined as serum B_12_ concentration <200 pg/ml, and folate deficiency was defined as red blood cell folate concentration <102.6 ng/ml (16). If there was evidence of iron, vitamin B_12_, and/or folate deficiency, the participant was defined as having a vitamin deficiency.

If there was no evidence of a vitamin deficiency, other determinants were queried. If estimated creatinine clearance was <30 ml/min (17), participants were identified as having chronic renal disease, and if they had serum iron <60 μg/ml but without evidence of iron deficiency, they were identified as having chronic inflammation, as described by Guralnik, et al. (5). Hemolysis was defined if a participant had total bilirubin >1.2 mg/dl and lactate dehydrogenase (LDH) >220 U/L. For each participant, prescription drug use was queried to identify those participants taking medications that can be associated with cytopenia (**Supplemental Table 1**). Pregnancy status at the time of the exam and history of hepatitis was also queried for each participant. Participants with a cytopenia and without one of the listed determinants were classified as having an UC for this analysis.

### Statistical Analysis

Prevalence rates of cytopenia and UC were estimated by applying the NHANES sampling weights to obtain national estimates for the US population eligible for the study. For those with cytopenia, the potential explanations for cytopenia available in NHANES and the type of cytopenia were examined. The distributions of hemoglobin, neutrophil count, and platelet count for those with UC were compared to the distributions of the overall NHANES population, by race and gender. Demographics for those with no cytopenia, explained cytopenia, and UC were compared using χ^2^ tests and univariable linear regression. Multivariable logistic regression was used to assess the characteristics associated with UC, compared to those with no cytopenia. All variables with p < 0.10 in the adjusted analysis were included in the final model. All analyses accounted for the complex sampling design of NHANES by incorporating the NHANES provided design variables and reported statistics represent weighted values. All analyses were conducted using SAS software, v9.4 (SAS Institute, Cary, NC).

## Results

### Overall Cytopenia Prevalence and Etiologies

There were 7,962 participants who met the selection criteria, corresponding to a weighted, nationally representative population of 170,864,876 persons. Two hundred and thirty-six participants (corresponding to a national estimate of 3,485,383 persons) had any cytopenia (2.0%). Overall, cytopenia was more common among women (2.5%) and Non-Hispanic Black (NHB) participants (7.1%).

Among participants with a cytopenia (n = 236), possible reasons for the cytopenia were examined. Similar to the approach used by Guralnik et al. (5), cytopenia was first classified by whether or not there was a concomitant nutrient deficiency. Approximately 44% of cytopenias were explained by a nutrient deficiency, while 13% could be explained by other causes, the most common of which was chronic inflammation. Forty-three percent (n = 86) were not explained by any of the listed reasons (**Table 1**). This corresponded to a prevalence of UC in the NHANES population ≥20 years of 0.9%.

**Table 1:** Distribution of possible determinants of cytopenia (n = 236).

	Raw Number	National Estimate	Percent (Type)	Percent (All Cytopenia)
**With Nutrient Deficiency**				
Iron deficiency	97	1,469,427	96.5	42.2
Vitamin B_12_ deficiency	3	18,884	1.2	0.5
Folate deficiency	0	0	0.0	0.0
Multiple nutrient deficiencies	7	34,937	2.3	1.0
*Total*	*107*	*1,523,248*	*100.0*	*43.7*
**Without Nutrient Deficiency**				
Renal insufficiency	6	62,224	3.2	1.8
Chronic inflammation	13	154,010	7.8	4.4
Medications	1	17,031	0.9	0.5
Pregnancy	7	21,823	1.1	0.6
Hepatitis	8	71,995	3.6	2.1
Hemolysis	1	10,519	0.5	0.3
Multiple non-nutrient reasons	7	124,360	6.3	3.6
Unexplained	86	1,500,173	76.5	43.0
*Total*	*129*	*1,962,136*	*100.0*	*56.3*
**All cytopenias**	**236**	**3,485,383**	**N/A**	**100.0**

### Race and Gender Distributions of Unexplained Cytopenia

**Figure 2:**
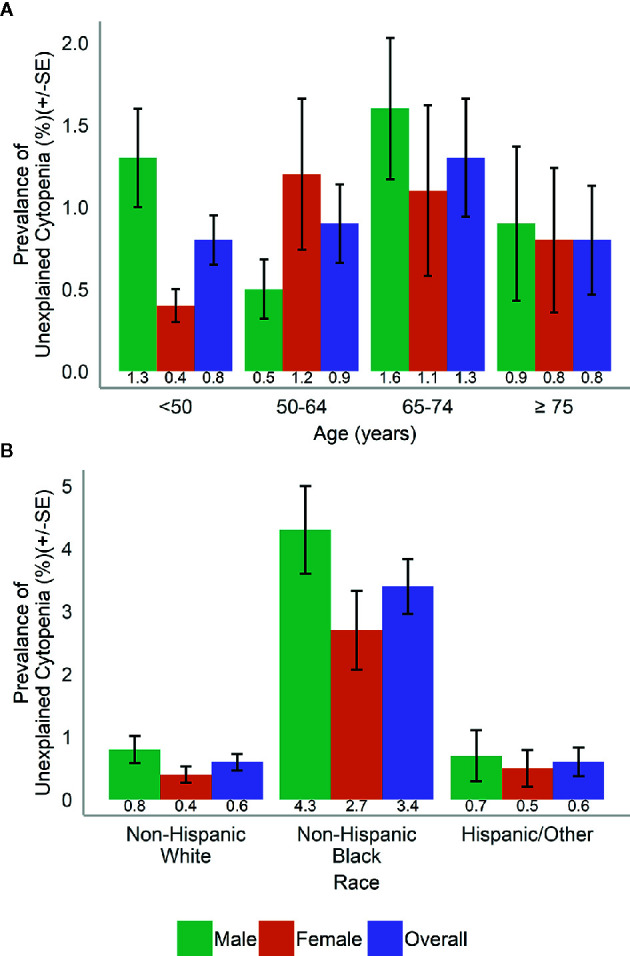
Prevalence of unexplained cytopenia by **(A)** age and gender and **(B)** race and gender.

In the population, UC was more common in men than in women (1.1 *vs*. 0.7%) and varied with age (**Figure 2A**). UC was most common in NHB (3.4%) compared to participants of other races and across both genders (**Figure 2B**).

Among those with UC, the most common type of cytopenia in both men and women was neutropenia alone (79.4 and 67.3%, respectively). In women, this was followed by anemia alone (20.8%) and thrombocytopenia alone (11.6%) and a combination of multiple cytopenias (0.3%). In men, thrombocytopenia alone was the next most common (12.2%) followed by anemia alone (5.2%) and a combination of multiple cytopenias (3.1%). When examined by race, almost all Hispanic/Other participants with UC had neutropenia alone (87.1%) compared to 79.5% of NHB and 68.1% of Non-Hispanic White (NHW) participants. NHWs were comparatively more likely to have thrombocytopenia alone (20.1%, compared to 4.1 and 4.3% for NHB and Hispanic/Other), while NHB participants had comparatively more cases of anemia alone (13.9%, compared to 9.9 and 7.7% for NHW and Hispanic/Other).

**Figure 3:**
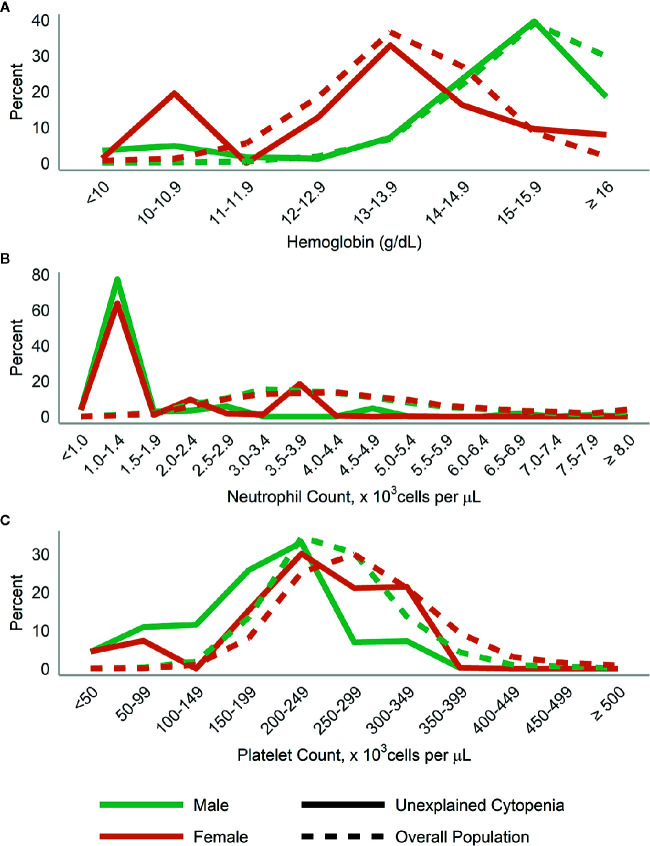
Distribution of **(A)** hemoglobin; **(B)** neutrophils; and **(C)** platelets in those with unexplained cytopenia, compared to the overall population, stratified by gender.

**Figure 4:**
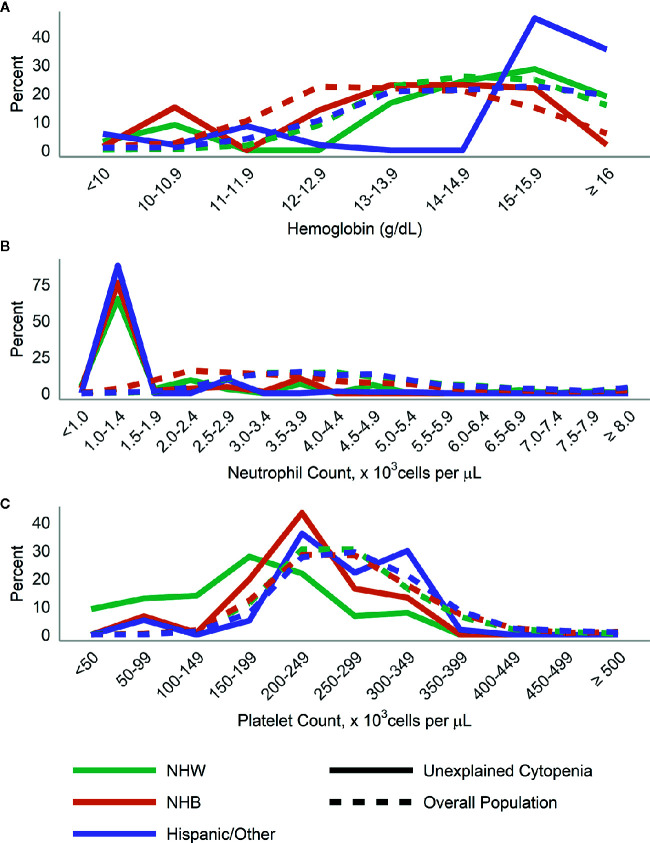
Distribution of **(A)** hemoglobin, **(B)** neutrophils, and **(C)** platelets in those with unexplained cytopenia compared to the overall population, stratified by race/ethnicity.

When we examined the distributions of blood levels according to gender (**Figure 3** **)** and race/ethnicity (**Figure 4**), across both race and gender a majority of those with UC had neutrophils <1,500 cells/µl. This was particularly true for Hispanic/Other participants, who at the same time had a large number of participants with high (≥15 g/dl) hemoglobin.

### Characteristics of Participants With and Without Unexplained Cytopenia

Compared to those with no cytopenia (n = 7726) and those with an explained cytopenia (n = 150), those with UC (n = 86) were significantly more likely to be male, NHB, and tended to be more likely to work in an office setting. There was no significant difference in age or body mass index (BMI). Those with explained and UC had significantly fewer alcoholic drinks per day, on average, and significantly fewer days with 5+ drinks in a single day compared to those without any cytopenia (**Table 2**).

**Table 2:** Demographics of the sample, by presence of a cytopenia.

	No Cytopenia (n = 7726)	Explained Cytopenia (n = 150)	Unexplained Cytopenia (n = 86)	*p-value*
**Variable**	n(weighted %)	n(weighted %)	n(weighted %)	
***Demographics***				
Age (years)				0.1231
<50	4,215 (63.1)	103 (68.7)	40 (59.7)	
50–64	1,659 (21.5)	14 (12.1)	19 (21.3)	
65–74	1,014 (8.9)	11 (5.8)	18 (12.9)	
≥75	838 (6.5)	22(13.3)	9 (6.1)	
Gender				<.0001
Male	3,726 (49.0)	28 (18.1)	51 (61.8)	
Female	4,000 (51.0)	122 (81.9)	35 (38.2)	
Race/Ethnicity				<.0001
Non-Hispanic White	3,833 (72.2)	35 (37.3)	24 (49.1)	
Non-Hispanic Black	1,321 (9.4)	53 (31.6)	48 (38.4)	
Hispanic/Other	2,572 (18.4)	62 (31.1)	14 (12.4)	
BMI (kg/m^2^)				0.5810
<18.5	124 (1.9)	1 (2.2)	4 (4.4)	
18.5–24.9	2,319 (32.9)	49 (27.5)	30 (39.6)	
25–29.9	2,783 (34.8)	51 (32.8)	29 (37.1)	
≥30	2,428 (29.7)	42 (32.5)	22 (18.1)	
*Missing*	72 (0.7)	7 (4.9)	1 (0.8)	
Occupational Group*				0.1484
Not Working	3,291 (33.6)	77 (37.4)	38 (32.5)	
Office	2,254 (38.2)	43 (40.6)	30 (48.3)	
Service	733 (8.9)	18 (13.3)	7 (6.3)	
Agriculture, ECR, OFL	1,437 (19.2)	11 (8.2)	11 (12.9)	
*Missing*	11 (0.2)	1 (0.4)	0 (0.0)	
***Alcohol Use***				
Ever a time where you had 5+ drinks every day?				0.1884
No	6291 (82.2)	127 (85.9)	72(86.8)	
Yes	1,060 (13.7)	16 (8.2)	12 (12.4)	
*Missing*	375 (4.1)	7 (5.9)	2 (0.8)	
Average number of drinks/day^ [Mean (SE)]	2.0 (0.06)	1.6 (0.26)	1.4 (0.27)	0.0199
Number of days in last year with 5+ drinks [Mean (SE)]	13.7 (0.69)	7.26(2.31)	6.0 (2.63)	0.0023

*Occupational group was defined based on the US Census Bureau’s Census Indexes of Industrial and Occupational Source Codes and then collapsed into the categories above.

^For those who did not drink at all in the last year, an average of 0 drinks per day was imputed.

BMI, Body Mass Index; ECR, Extractive, Construction, and Repair; OFL, Operators, Fabricators, and Labor.

p-values based on non-missing values.

The final adjusted model of characteristics associated with presence of UC compared to those with no cytopenia included gender, race/ethnicity, BMI, occupational group [as defined by Liu and Jia (18)], and average number of drinks per day (all adjusted p-value < 0.10). Those with UC were significantly less likely to be female (Adjusted Odds Ratio (AOR):0.47, 95% Confidence Interval (CI): 0.27–0.81), have BMI ≥30 kg/m^2^, compared to 18.5–24.9 kg/m^2^ (AOR:0.41, 95% CI: 0.22–0.78), and to be working in service jobs (AOR:0.43, 95% CI: 0.19–0.98) compared to office jobs. They were significantly more likely to be NHB, compared to NHW (AOR:7.00, 95% CI: 4.45–11.00). Although those with an increase in reported number of drinks per day tended to be less likely to have UC, these results were not statistically significant (p = 0.0686) (**Table 3**).

**Table 3:** Independent factors associated with presence of unexplained cytopenia compared to no cytopenia.

	Unexplained Cytopenia *vs.* No Cytopenia
***Variable***	AOR* (95% CI)
Gender	
Female *vs*. Male	0.47 (0.27–0.81)
Race/Ethnicity	
Non-Hispanic White	1.0 (ref)
Non-Hispanic Black	7.0 (4.45–11.00)
Hispanic/Other	1.25 (0.49–3.21)
BMI (kg/m^2^)	
18.5–24.9	1.0 (ref)
<18.5	1.99 (0.54–7.28)
25–29.9	0.83 (0.39–1.78)
≥30	0.41 (0.22–0.78)
Occupational Group	
Office	1.0 (ref)
Service	0.43 (0.19–0.98)
Agriculture, ECR, OFL	0.43 (0.13–1.45)
Not Working	0.66 (0.41–1.07)
Average Number of Drinks Per Day	0.84 (0.70–1.01)

*adjusted for all variables listed.

AOR, Adjusted Odds Ratio; CI, Confidence Interval; BMI, Body Mass Index; BMI, Body Mass Index; ECR, Extractive, Construction, and Repair; OFL, Operators, Fabricators, and Labor.

n = 7,350.

## Discussion

This analysis indicates that the overall prevalence of UC in a US nationally representative sample is approximately 1%. To our knowledge, this is a new finding and the first report of the prevalence of UC within the general population. To date only one study has detailed the prevalence of unexplained anemia using a large population-based study such as NHANES (5). However, this study did not explore the prevalence of unexplained neutropenia or thrombocytopenia and limited analysis to those at least 65 years old. Although the estimated prevalence in the current analysis was low, it provides valuable insight into patterns of UC in the general population, as NHANES generally surveys healthy people as opposed to hematology clinics which normally survey patients who show up with reported symptoms.

Detecting UC is important due to the potential for an eventual diagnosis of MDS. Demographic predictors for the development of MDS include older age, male sex, and white race (19, 20). Though not all findings of incident UC will lead to diagnosis of MDS, further evaluation and testing are warranted on those with UC to determine the cause, if any. Thus, identifying the populations in which UC poses the greatest risk of developing into a clinically relevant hematological disorder is a point of interest.

In the present analysis, elderly study subjects had the highest prevalence of UC although this prevalence was not statistically significantly different from what was observed in subjects with explained cytopenia. This is in agreement with prior studies, such as Guralnik and colleagues, who have indicated that anemia becomes more common in elderly individuals, with idiopathic anemia comprising approximately 30–40% of such cases (5, 21). Our study used a stricter definition for anemia (11 g/dl for both men and women) in comparison to the study by Guralnik et al., and thus the absolute rates are not comparable between the two studies. When data from other developed nations across the globe are reviewed, prevalence rates of anemia appear similar to our findings (22, 23). However, these studies do not characterize the nature or contemporary presence of neutropenia or thrombocytopenia.

Subjects with UC were more likely to be NHB. Extensive research has further described increased prevalence of anemia, neutropenia, and thrombocytopenia amongst those of non-white race/ethnicity (24), particularly African Americans, who have lower blood counts independently from socioeconomic and nutritional factors (15, 25). The consistent difference by race seems to indicate some genetic characteristics that define the blood count. Genomic research has become the new focus in determining incident or undifferentiated neutropenia. There appear to be specific genomic factors that result in benign ethnic neutropenia (26), particularly the Duffy Antigen Receptor for Chemokines (DARC) gene null red phenotype, which leads to neutrophil migration to the spleen, thus resulting in a relative neutropenia. Globally, females have been shown to have a slightly higher prevalence of anemia (27), but to our knowledge, research on sex differences in neutropenia and thrombocytopenia is limited. In our analysis subjects with explained cytopenia were more likely to be female, but subjects with UC were more likely to be male. Further exploration into the occurrence of unexplained pancytopenia and possible resultant ICUS based on sex may be warranted.

Results should be interpreted within the context of limitations of the data and analysis. As the raw number of participants with UC is relatively low, there is higher variability in the prevalence estimates. Analysis was limited to 1999–2002 based on the availability of necessary measures in NHANES, so future research may benefit from more current data. Additionally, only one measurement per individual was available, since NHANES is a cross-sectional study. Participants are not followed over time, so the biospecimens collected are representative only of a single event. Values detected in this analysis could be indicative of a benign or chronic condition, persistent UC, or possible MDS. Diagnosis of conditions such as ICUS requires multiple follow-up evaluations and confirmatory test such as bone marrow biopsy (1), which were not available in NHANES. Subsequent evaluations of those with UC with precision medicine techniques (28) such as next generation sequencing can assist in further differentiating UC as an incident lab finding, an inherited condition, or CCUS/ICUS. Although NHANES alerts subjects to abnormal values and provides guidance to participants on follow-up care with their own physicians, those results are not reported back to NHANES.

Although blood cell values fluctuate and could be modified by viral infections or medications, we attempted to take this into account by excluding from the analysis subjects who reported medications and chronic illness known to influence blood count. However, it is important to note that participants in NHANES were evaluated on these variables *via* self-report, and therefore, we cannot completely discount the presence of unreported or undetected infections or the use of other medications. Additionally, we were unable to conduct full clinical procedures to rule out other determinants of cytopenia, and there may be some that are not captured in NHANES. As a result, some participants classified as having UC, may also have an unmeasured determinant, so we may overestimate the prevalence of UC in the population. However, we believe that we captured the most prevalent explanations for a cytopenia and that estimates provide a valuable baseline for an understudied condition.

## Conclusion

This analysis provides the first estimates of population level prevalence of UC in a nationally representative cohort and represents a baseline reference in the overall US population. Surveillance of UC is important, given the increased risk of hematologic malignancies in those with ICUS and CCUS. In order to further refine the characterization of these patients and prevalence estimates of conditions like ICUS and CCUS in the general population, future research should include more current data, as well as longitudinal studies to track patients with UC for eventual diagnoses.

## Data Availability Statement

Publicly available datasets were analyzed in this study. This data can be found here: https://wwwn.cdc.gov/nchs/nhanes/Default.aspx.

## Ethics Statement

The NHANES protocol is reviewed annually by the National Center for Health Statistics Ethics Review Board.

## Author Contributions

Conceptualization: ET and ES. Data curation and formal analysis: NA. Writing: Original draft preparation: NA, JR, ET and DT. Writing: Review and editing: NA, JR, JM, ES, DT, BM and ET. All authors contributed to the article and approved the submitted version.

## Funding

This research was partially funded by the National Cancer Institute (P30CA196521).

## Conflict of Interest

The authors declare that the research was conducted in the absence of any commercial or financial relationships that could be construed as a potential conflict of interest.
