# Supplementary material for: Prevalence of Cytopenia in the General Population—A National Health and Nutrition Examination Survey Analysis
Source: Front Oncol. 2020 Nov 20;10:579075. doi: 10.3389/fonc.2020.579075 (PMC7714991; doi:10.3389/fonc.2020.579075)
Supplement: Supplementary file 1 [file Table_1.docx]

S1 Table: List of prescription medication used to define a medication related cytopenia

| **Generic Drug Name** |
| --- |
| Bendroflumethiazide |
| Carbamazepine |
| Heparin |
| Hydralazine |
| Hydralazine; Hydrochlorothiazide |
| Hydralazine; Hydrochlorothiazide; Reserpine |
| Isoniazid; Rifampin |
| Methotrexate |
